# Current trends in nano-delivery systems for functional foods: a systematic review

**DOI:** 10.7717/peerj.14980

**Published:** 2023-03-17

**Authors:** Emmanuel Otchere, Brighid M. McKay, Marcia M. English, Alberta N. A. Aryee

**Affiliations:** 1 Department of Human Ecology, Delaware State University, Dover, Delaware, United States; 2 Department of Human Nutrition, St. Francis Xavier University, Antigonish, Nova Scotia, Canada

**Keywords:** Nanotechnology, Nano-delivery systems, Functional foods, Bioactive components

## Abstract

**Background:**

Increased awareness of the relationship between certain components in food beyond basic nutrition and health has generated interest in the production and consumption. Functional foods owe much of their health benefits to the presence of bioactive components. Despite their importance, their poor stability, solubility, and bioavailability may require the use of different strategies including nano-delivery systems (NDS) to sustain delivery and protection during handling, storage, and ingestion. Moreover, increasing consumer trend for non-animal sourced ingredients and interest in sustainable production invigorate the need to evaluate the utility of plant-based NDS.

**Method:**

In the present study, 129 articles were selected after screening from Google Scholar searches using key terms from current literature.

**Scope:**

This review provides an overview of current trends in the use of bioactive compounds as health-promoting ingredients in functional foods and the main methods used to stabilize these components. The use of plant proteins as carriers in NDS for bioactive compounds and the merits and challenges of this approach are also explored. Finally, the review discusses the application of protein-based NDS in food product development and highlights challenges and opportunities for future research.

**Key Findings:**

Plant-based NDS is gaining recognition in food research and industry for their role in improving the shelf life and bioavailability of bioactives. However, concerns about safety and possible toxicity limit their widespread application. Future research efforts that focus on mitigating or enhancing their safety for food applications is warranted.

## Introduction

Functional foods have gain prominence among consumers due to their perceived/demonstrated positive effects on health beyond basic nutrition (Peighambardoust et al., 2021; Topolska, Florkiewicz & Filipiak-Florkiewicz, 2021; Zhang et al., 2021). The rapidly expanding functional food industry in the United States is expected to continue at a compound annual growth rate of 3.5% (2021–2025) (Agriculture & Agri-Food Canada, 2022). This expansion is linked to increasing interest in health and wellness and purchasing trends such as consumption of immune-boosting foods during the COVID-19 pandemic (Agriculture & Agri-Food Canada, 2022; English, 2022).

The bioactive compounds which make the food “functional” provide physiological benefits such as antioxidant, anti-inflammatory and anti-cancer properties and reduce the risk of non-communicable diseases (Adefegha, 2018; Topolska, Florkiewicz & Filipiak-Florkiewicz, 2021). Dietary intake of lipid or protein-derived bioactive compounds has been linked with the reduction of chronic diseases (Zhang et al., 2021). Although studies demonstrate the potential of these health-promoting components, their stability during production and when consumed, direct application into food products and controlled release at the target site still remains a challenge (Zhang et al., 2021).

Innovative technologies that address structure, stability and degradation, and sensory attributes is an active area of research (Huang et al., 2017b; McClements & Öztürk, 2021; Peighambardoust et al., 2021). This includes nanotechnology as a promising solution, through which the bioactive components are encapsulated, protected, and delivered with improved efficiency and efficacy (McClements & Öztürk, 2021). This has also been shown to enhance the biocompatibility and bioavailability of the encapsulated material (Jones, Caballero & Davidov-Pardo, 2019; Pateiro et al., 2021). However, this strategy may pose safety concerns, such as the potential of allergenic or toxic peptides and proteins (Liu et al., 2021; Peighambardoust et al., 2021), and may require regulatory oversight; particularly if the materials lack human consumption studies records. Additional concerns relates to consumption—frequency, duration, dosage to observe their beneficial physiological activity (Peighambardoust et al., 2021). Several forms of nanotechnology including nanoencapsulation have been described. The core material is encased using solid or liquid wall material (Rehman et al., 2019; Reque & Brandelli, 2021). The formed may be nanocapsules, nanospheres, or solid-lipid nanoparticles (SLNs). Nanocapsules are vesicular systems containing a cavity with an inner liquid core that encapsulates the active ingredient (Rehman et al., 2019), whilst nanospheres are formed when the active core is enclosed by a shell, membrane, or coating. Nanospheres are matrix systems used to uniformly disperse active ingredients (Rehman et al., 2019). In principle, nanocapsules must be smaller than 1000 nm, however cosmetic and pharmaceutical regulations require them to be less than 100 nm (Assadpour & Jafari, 2019; Rehman et al., 2019). During nanoencapsulation, small sized core materials (10–100 nm) are directed to targeted off-loading sites. Previous studies explored the merits of polymer-based nano-delivery systems (NDS) including the use of polysaccharides, lipids, and proteins. Polysaccharides are used for their bio-adhesive properties (Hu et al., 2017), while lipid-based NDS include nanoliposomes, SLNs and nanostructured lipid transporters (NLCs) are used for their biocompatibility, loading capacity, solubility, and bioavailability properties. Other NDS include nanoemulsions, nanogels and pickering emulsions, a type of NLC (Livney, 2015; McClements & Öztürk, 2021).

Although the application of nanotechnology in functional food products is not widespread, far fewer studies of protein-based NDS exist. The use of protein-based NDS may offer additional advantages due to their diverse functional properties. The polar, non-polar and or charged functional groups of proteins, and amphoteric and electrical properties allow their interaction with various bioactive substances (Lin et al., 2021; Rajendran, Udenigwe & Yada, 2016) rendering proteins as effective carriers. Their unique structure and high affinity for hydrophobic sites make proteins ideal carriers for bioactives (Zhang et al., 2021), and amphiphilicity for encapsulating non-polar amino acids and other components. Consequently, when protein-based nanocarriers transport non-polar amino acids, they may form hydrophobic bonds, which facilitates encapsulation (Lin et al., 2021).

This review aims to examine recent methods used to stabilize bioactive components, and will specifically explore the use, advantages, and challenges of plant proteins as carriers in NDS for bioactive compounds, and their application in food product development.

## Survey methodology

### Search and selection of studies

All articles were selected from the Google Scholar database by using the specific search string “Nano-delivery systems”. An advanced search was done by introducing filters such that, all the articles should have the words “Nano-delivery”, with an exact phrase of “food”, with at least one word of “Types of nano-delivery systems” OR “Protein-based nano-delivery systems” OR “challenges of protein-based nano-delivery system” OR “Benefits of protein-based nano-delivery systems”, and these filters with words can occur in any part of the article. In addition, the years of publications for the articles was customized and limited to 2015–2023 to get current information about NDS. No filters were applied for the authors, publication titles and the type of the articles. The last date for completing the search was January 6, 2022.

All the articles were downloaded, and two authors carefully read the abstracts of all the articles to pre-screen the selected articles. The remaining sections of the selected articles were read by the same authors. Other references cited in these articles not part of the main search but were related to the scope of this review were assessed and included in this review if they met the eligibility criteria. All misunderstandings between the two authors with regards to the inclusion or exclusion of an article in the final review were sent to the two other authors for further verification.

### Eligibility criteria

**Figure 1:**
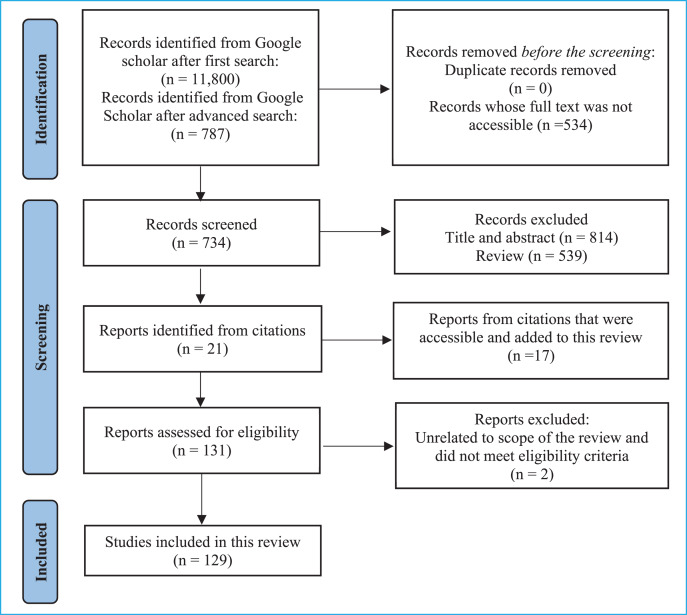
Flow diagram of information search for the systematic review (Page et al., 2021).

A total of 11,800 articles were obtained for the first search. This was reduced to 787 articles after applying the filters for the advanced search (Fig. 1). The screening of the abstracts and title was done to select suitable articles for this review with a focus on NDS in foods, and finally 129 articles were included in this review.

### Data extraction

After reading all the selected articles, data from the articles were categorized into subtopics. Data about each subtopic including types on NDS, applications of NDS in developing new foods, and the challenges and opportunities for future research with NDS were sourced from all selected articles. In addition, reported data from several articles were extracted to design a table in the present review.

### Data synthesis

The extracted data were synthesized into a narrative form under various categories including an overview of NDS, types, and benefits, and the applications and challenges of using NDS in food.

### Risk of bias assessment

The risk of bias was assessed with 10 questions, which was answered as yes, can’t tell or no, according to the checklist provided by Critical Appraisal Skills Program (CASP) for systematic reviews. An independent assessment of all articles was done by two authors and only articles which received a minimum of eight yes out of ten, indicating a good quality were included in the review.

## Overview of nano-delivery systems

Nanotechnology is an emerging field widely recognized in the food industry to potentially be an effective delivery system for functional foods. Nano-delivery systems (NDS) enriched core of nutraceuticals, including vitamins, bioactive peptides, antioxidants, or probiotics and shell/wall materials at submicron sizes (1–200 nm) with greater surface area enhance their role as vehicles, and protect the core from degradation, and improve their stability, solubility, effective delivery, and bioavailability to targeted sites (Ha et al., 2019).

## Types and benefits of nano-delivery systems

Several types of NDS are universally used in the food industry to deliver bioactive compounds in food products. Recent reports of bioactives and their respective nanocarriers and food applications are highlighted in Table 1, showing wide range of nanocarriers, with specific functions and benefits. For instance, nanocarriers improved the stability and bioavailability of certain vitamins, such as folic acid and vitamin D, while nanoemulsions enhanced the release of carotenoids, including β-carotenes, within the intestine, and improved the cargo’s solubility and antioxidant activity (Sampathkumar, Tan & Say, 2020).

**Table 1:** Recently reported nano-delivery systems for bioactives in foods.

Classification	Applications	Bioactive component	Nanocarrier	References
Vitamins	Clear beverage products, fruit juices	A	SLN; NLC; Nanocomplex; Nanosuspension	Salminen et al. (2016), Resende, Costa Lima & Reis (2020), Ghasemi & Abbasi (2014), Campardelli & Reverchon (2015)
		Folic Acid (B9)	Nanodroplets in calcium alginate; Mesoporous silica particle	Bakhshi et al. (2013), Ruiz-Rico et al. (2017)
		D	Soybean β-conglycinin NPs	Levinson, Israeli-Lev & Livney (2014)
		D2	Caseinate nanocomplex	Tan et al. (2014)
		D3	Protein-polysaccharide nanocomplexes; NLC; MUFA-emulsions	Xiang et al. (2020), Mohammadi et al. (2017), Schoener et al. (2019)
		E	Nanoemulsions	Sahafi et al. (2021), Dima et al. (2020), Manocha et al. (2022)
Mineral	Baked products (bread)	Calcium	Whey protein hydrolysate nanocomposite	Xixi et al. (2015), Dima et al. (2020)
Carotenoids	Yogurt and dairy products	β-carotene	Nanoemulsions in SLN; Protein-stabilized SLNs; Reassembled casein micelles	Salminen et al. (2016), Borba et al. (2019), Mehrad et al. (2018), Rao et al. (2013)
		Bixin	Nanocapsules	Lobato et al. (2015)
		Lutein	Nanoemulsions stabilized with food grade; Nanoemulsifiers	Sedaghat Doost et al. (2020)
		Lycopene	Nanoemulsifiers; Liposomes	Tan et al. (2014), Alu’datt et al. (2022), Falsafi et al. (2022)
		Astaxanthin	Food-grade pickering emulsion	Burgos-Díaz et al. (2020)
Polyphenols	Yogurt, high fat content foods, blueberries, grapes, and soybean oil	Curcumin	Casein NPs; Phosphocase in micelles; SLN and NLC	Pan, Zhong & Baek (2013), Benzaria et al. (2013), Huang et al. (2020), Ban et al. (2020), Pinheiro, Vicente & Gonçalves (2023)
		EGCG	β-Lactoglobulin complexes; Lactoferrin-based NPs; Liposomes; Chitosan-based NPs; Casein micelles	Shpigelman, Cohen & Livney (2012), Khan et al. (2014), Haratifar, Meckling & Corredig (2014), Alu’datt et al. (2022)
		Quercetin	Lecithin/chitosan NPs; Nanoemulsion; Co-encapsulation with ALA in NLC	Souza et al. (2014), Pool et al. (2013), Huang et al. (2017b)
		Resveratrol	Nanoemulsions; Liposomes	Davidov-Pardo & McClements (2014), Lemes et al. (2017), Ajeeshkumar et al. (2021)
		D-limonene	Encapsulated SLN; Nanoemulsions; nanosuspensions	Akhavan-Mahdavi et al. (2022)
		Catechins	Lipid-based nanoparticles; nanoemulsions; polymeric nanoparticles	Rashidinejad et al. (2021), Liu et al. (2017)
		Luteolin	Soybean protein isolate based NDS	Sun et al. (2022), Estakhr et al. (2020)
Fatty acids	Meat products and baked products	DHA	Casein micelles and Nanoparticles; B-lactoglobulin-pectin nanodispersion Nanoliposomes	Zimet & Livney (2009), Rasti et al. (2012)
		Omega 3	SLN and Nanoemulsion; NLC; Casein micelles	Salminen et al. (2016), Ghasemi & Abbasi (2014)
		EPA	Nanoliposomes	Rasti et al. (2012)
Antioxidant	Fruit juices	ALA; α-tocopherol	Nanoemulsion; Co-encapsulation with quercetin in NLC	Liu et al. (2021), Huang et al. (2017a), Dima et al. (2020)
Phytosterol	Butter	β-sitosterol	NLC	Bagherpour et al. (2017)

NDS used in food include both nanocapsules and nanospheres, and can be lipid-, protein-, or polysaccharide/poly acid-based. Lipid-based NDS have been thoroughly studied and are highly advantageous when compared to the other encapsulation strategies (Luykx et al., 2008). Lipid-based systems include liposomes, nano-emulsions, solid-lipid nanoparticles (SLNs), and nanostructured lipid carriers (NLCs). SLNs and NLCs are quite common and were developed to address issues related to previous NDS (Naseri, Valizadeh & Zakeri-Milani, 2015). Liposomes are used for their ability to deliver hydrophilic and lipophilic bioactives, although they are extremely sensitive to environmental conditions, (Livney, 2015). Nano-emulsions, specifically oil-in-water nano-emulsions, have better physical stability and bioavailability when compared to conventional emulsions, due to their small size and large surface area (Lin et al., 2021). SLNs are similar to nano-emulsions as they are colloidal dispersions of small liquid droplets (Livney, 2015) but their dispersions solidify in oil. Specifically, the molecules within SLNs are organized into crystalline structures (McClements & Öztürk, 2021) that prevent leakage of the bioactive ingredient (Assadpour & Jafari, 2019). Being made of natural or synthetic lipids (Reque & Brandelli, 2021), they contain hydrophilic shells with the hydrophobic lipid core that facilitates the control of the size and release profile of the bioactive ingredients (Cerqueira et al., 2014; Reque & Brandelli, 2021). Conversely, SLNs have reduced chemical stability due to their fully crystallized dispersion phase (Livney, 2015; McClements & Öztürk, 2021). To address these issues, NLCs were designed with partially crystalized nanovesicle particles dispersed in aqueous phase with an emulsifier (Livney, 2017). NLCs offer increased stability and the ability to encapsulate more bioactives (Reque & Brandelli, 2021) compared to SLNs.

NDS enables the enrichment of food and beverages with bioactives without compromising their sensory attributes (Livney, 2017). The nanometric size of the particles allows their incorporation without negatively affecting the sensory attributes. For instance, fortification with omega-3 fatty acids is often challenging as these compounds oxidize easily, resulting in fish-like off-flavors (Ha et al., 2019). Studies showed whey-protein based nanoparticles and nanoemulsions of omega-3 fatty acid with reduced off-flavor (Ha et al., 2019; Huang et al., 2017a). NDS has been used as a stabilization strategy to improve food storage (Ha et al., 2019), handling (McClements & Öztürk, 2021) and safety while minimizing chemical and physical changes. NDS have been shown to facilitates the transport of bioactives in functional foods, inhibit bioactives cargo degradation during digestion and transit through the gastrointestinal tract, thereby improving stability, effective delivery and bioavailability (McClements & Öztürk, 2021).

## Protein based nano-delivery systems

Protein-based NDS show promise as delivery mechanisms within the food industry (Lin et al., 2021). Proteins used in NDS are mostly derived from microbial, animal, and plant sources. This includes casein, whey, gelatin, albumin, silk, zein, gliadin, soy, and pea (Dai et al., 2019; Stevenson, Lewis & Whittington, 2018; Takeuchi et al., 2019; Tari et al., 2018). Recent increase in interest in plant-based protein NDS relates to their lower susceptibility to pathogenic infections compared to animal-sourced materials (Maviah et al., 2020), suitability for those who follow certain religious practices or vegan diet, and general environmental sustainability.

The adaptability and versatility of the chemical composition, and various functional groups in protein, form several complexes with polysaccharides, lipids, or other biopolymers allowing their interaction with a diverse group of bioactive compounds and nutrients, and increase their potential application in food (Ha et al., 2019; Lin et al., 2021). The various molecular and physicochemical properties of proteins, such as size, shape, charge, surface properties, water dispersibility, colloidal stability and stability, determine their utility (Puttasiddaiah et al., 2022; Zhou et al., 2020). The versatile composition of proteins affords several interactions including covalent, electrostatic, and hydrophobic. The latter is attributed to their amphiphilic properties. The polar and non-polar amino acid residues, and functional groups facilitate the incorporation of bioactives more easily *via* primary amino groups or sulfhydryl groups (Luykx et al., 2008) rendering proteins as suitable carriers. These interactions also contribute to the unique features of protein-based NDS, such as their ability to control retention and release of cargo (Zhang et al., 2021). As such, protein-based NDS have the important ability to release their payload in response to specific environmental triggers, such as pH, ionic strength, temperature, enzyme activity or redox conditions. These factors are important for stimulus responsive protein nanocarriers involved in preventing the premature release of cargo to improve “utilization rate of cargo” (Zhang et al., 2021). This function can be beneficial in pharmaceuticals, but also in the delivery of bioactives in food products. Protein-based NDS also possess unique functional properties, such as gelation and emulsification, ideal for encapsulating bioactive compounds (Luykx et al., 2008).

Protein-based NDS can be produced using a variety of techniques which can simple and cost-effective, including coacervation, gelation, spray drying, and electro-hydrodynamic processes (Fathi, Donsi & McClements, 2018; Puttasiddaiah et al., 2022). The selection of method is dependent on several factors including availability of materials and equipment, stability, compatibility, release mechanism and conditions, biodegradability, economic feasibility, application, function to be enhanced, and storage (Fathi, Donsi & McClements, 2018; Martínez-Álvarez, Calvo & Gómez-Estaca, 2020; Rajendran, Udenigwe & Yada, 2016). Gómez-Guillén et al. (2018) reported improved functionality and bioavailability of astaxanthin encapsulated by spray drying. Fathi, Donsi & McClements (2018) did not find gelation suitable for heat-sensitive cargo. Several studies have examined the use of whey proteins from animal sources in NDS for food due to their positive impact on texture and quality (Ha et al., 2019; Liu et al., 2021; Sampathkumar, Tan & Say, 2020). Whey proteins have been used to incorporate vitamins such as folate (Sampathkumar, Tan & Say, 2020) and folic acid, β-carotene, and caffeine (Liu et al., 2021) in NDS as nanosuspensions, solid dispersions, nano-emulsions, and hydrogels (Farooq et al., 2019; Maviah et al., 2020).

The preparation of NDS can modify the properties of the protein and influence their functionality making it important to understand the changes that occur during these processes. One of the limitations in the use of proteins in NDS is denaturation, and interfacial affinity which can induce protein adsorption and immobilization (Rajendran, Udenigwe & Yada, 2016; Relkin, Shukat & Moulin, 2014; Santiago et al., 2008). This area of research, related to understanding the chemical interactions, would benefit from further explorations to facilitate widespread adoption of protein-based NDS. Expansion of this area would also provide information on the mechanisms involved in transporting bioactives and would benefit the safety aspects related to their incorporation into food products.

## Applications of nano-delivery systems in food product development

**Figure 2:**
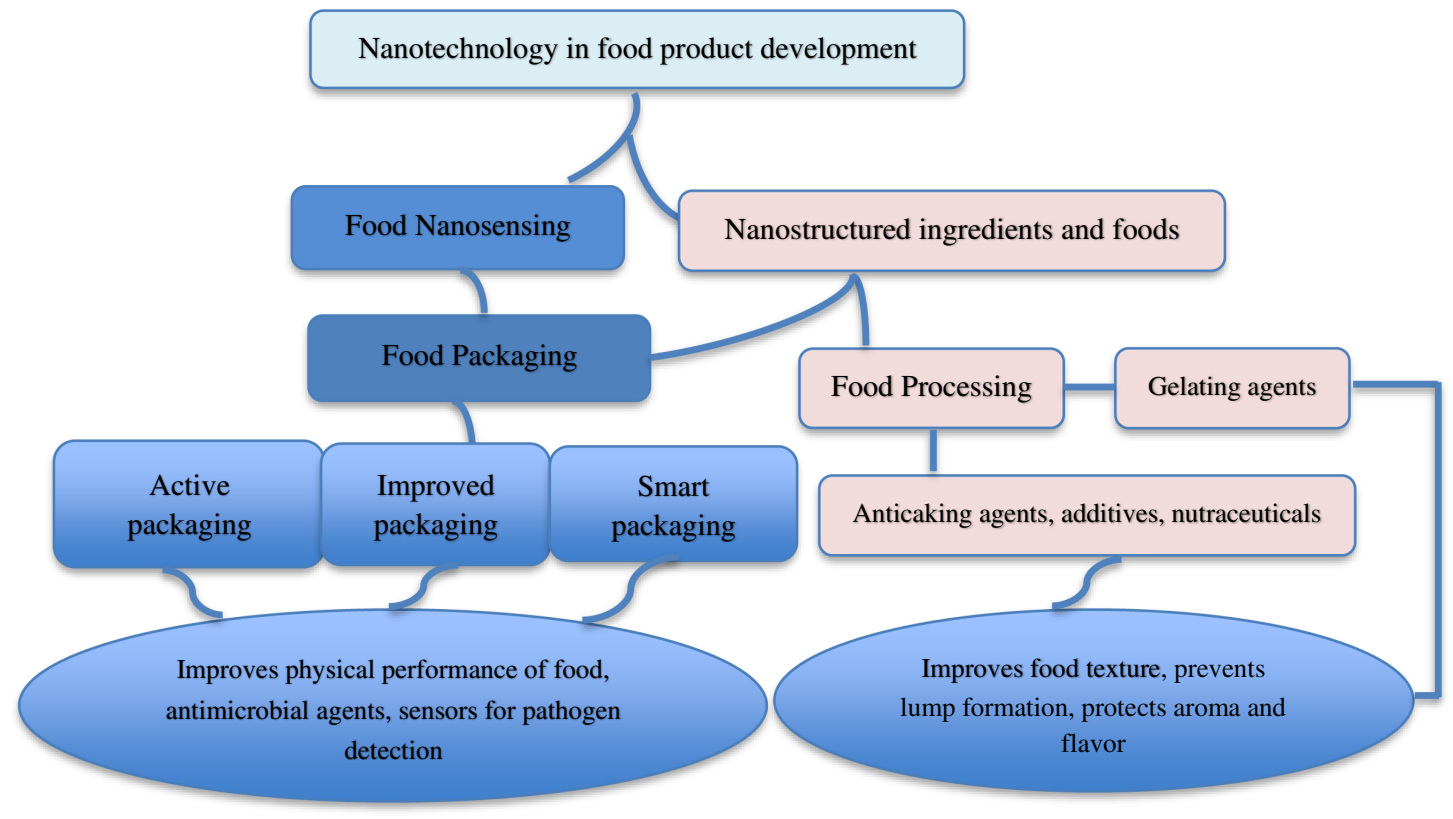
Application of nanotechnology in food product development.

The application of nanotechnology is expanding in the food industry as major food companies, including Heinz, Nestle and Kraft who are investing in nano-enabled foods and food packaging (Momin & Joshi, 2015; Sampathkumar, Tan & Say, 2020). Figure 2 shows the examples of application of nanotechnology in food product development. Numerous applications of nanotechnology in food packaging, functionality and processing exist, including nanoencapsulation, nanosensors, nanopacking, and smart distribution systems (Fadiji et al., 2022). Nestlé’s low-fat ice cream uses nanoemulsions to achieve a low-fat content (Sampathkumar, Tan & Say, 2020). These nanomaterials, as shown in several studies, enhance food packaging effectiveness, shelf life, and nutritional value without altering the flavor and physical properties of the food (Bajpai et al., 2018; Paul Das et al., 2018). NDS has also been successfully used as processing aids, quality enhancers, antimicrobial agents, as well as in the creation of sensors for evaluating quality, safety and enhancement of the product’s physicochemical characteristics, nutrition and bioavailability (Patra, Shin & Paramithiotis, 2018). Their application in the food industry can be categorized into two primary areas: nanostructured ingredients and nanosensing (Shafiq et al., 2020). Nanostructures can be utilized as carriers of food additives, intelligent nutrients, anti-caking agents, antibacterial agents, and fillers to increase the material’s mechanical strength and durability (Singh et al., 2017). For instance, milk, fermented cheese, and dairy desserts, are effective carriers for delivering probiotic bacteria in food. Milk and milk fats ensure the survival of probiotics by acting as a buffer to neutralize the harsh gastrointestinal conditions (Siciliano et al., 2021). Ranadheera et al. (2017) reported high acid tolerance level of ice cream and yogurt which ensured the survival of probiotics. Nanosensing has been used to improve the evaluation of food quality and safety (Luo et al., 2020). Nanoemulsion enhanced bioavailability of fat-soluble nutrients such as carotenoids by improving solubility, passive diffusion rate, and direct uptake by the intestine lymphatic system (Kanugo et al., 2022).

The application of nanotechnology in food packaging reveals a wide range of potential commercial uses (Han et al., 2018; Kumar et al., 2018; Mihindukulasuriya & Lim, 2014; Neme et al., 2021) in active, smart, and improved packaging. Active packaging as defined by the European regulation (EC) No. 450/2009 is a packaging system that interacts with the food by “deliberately incorporating components that would release or absorb substances into or from the packaged food or the environment surrounding the food” (Yildirim et al., 2018). It usually employs the use of metals such as silver (Ag), gold (Au), copper (Cu), and titanium dioxide (TiO_2_) because of their unique semi-conducting properties, which makes them a good fit as an adsorbent material, stain and catalytic substrate (Lin et al., 2021; Neme et al., 2021). Smart packaging employs nanoparticles to track microbiological, chemical, or biochemical growth inside food or in the environment around the product (Ashfaq et al., 2022). Nanoparticles are used in food packaging to retard the growth of microorganisms that may be present on food surfaces (Rossi et al., 2017). For instance, for food and beverages preservation, Ag-nanoparticles has been utilized as antimicrobial agent (da Costa Ribeiro et al., 2019; Fadiji et al., 2022). Arfat et al. (2017) used biocomposite film from fish skin gelatin and Ag-Cu nanoparticles to investigate the effect of Ag-Cu NPs loading on the thermal and antibacterial properties of gelatin-based film. The results indicated that the films improved thermal stability and antibacterial effect against gram-positive (L. *monocytogens*) and gram-negative (S. *thyphimurium*) bacteria. Additionally, Ashfaq et al. (2022) reported microbial growth inhibition when metal and metal oxide nanoparticles were used due to increased production of reactive oxygen species (ROS), which resulted in oxidative stress and cell death, and also by binding with DNA protein enzymes which interferes with cell function.

NDS is also currently use in nutraceuticals to manage and prevent diseases. The stability and bioavailability of curcumin, green tea, caffeine, eugenol improves their therapeutic functionality in the body. Zerumbone, first extracted from wild ginger is known for its numerous therapeutic effects, including anti-inflammatory, antibacterial, antiplatelet, antifungal, cytotoxic, and chemo preventive properties (Haque, Jantan & Harikrishnan, 2018). However, its low solubility in water and oral bioavailability limits its functionality (Md et al., 2018). With advancement in NDS, the poor oral bioavailability of zerumbone has been successfully improved. Several studies (Dorrington & Fraser, 2019; Foong et al., 2018; Md et al., 2018) have reported an increase in oral bioavailability and therapeutic effects of zerumbone when delivered *via* a NDS. In addition to the role of curcumin as a food additive and coloring agent, its anti-inflammatory property has been reported (Clarence et al., 2022). However, its application in pharmaceuticals was limited by its low solubility. Le et al. (2021) encapsulated curcumin in silica-containing redox nanoparticles and the results shows an improved solubility in water and anti-inflammatory effect, confirming an earlier report by McClements (2020).

Food product development is also benefiting from nanotechnology as the nanomaterials can be integrated into foods matrices without altering the sensory attributes of the products. In fact, the application of these smaller matrices into non-solid and semi-solid foods is necessary to reduce their impact on sensory properties (Luykx et al., 2008). Protein hydrogels are convenient and widely used in food applications (Luykx et al., 2008).

Literature searches reveal limited studies on the use of plant-based protein NDS in food products. Zein protein found in corn, which is primarily comprised of non-polar amino acids, has hydrophobic properties and is soluble in aqueous-alcohol solutions (Maviah et al., 2020). These properties as well as its gelling and adhesive properties make zein proteins an ideal nanocarrier for hydrophobic and hydrophobic biomolecules (Maviah et al., 2020). Although zein protein and other plant-based proteins are suitable nanocarriers, they are primarily used for drug delivery rather than food. This may prompt future studies due to their attractive properties and their positive impact on the texture and quality of food products. Plant-based proteins are widely used in colloidal delivery (Maviah et al., 2020). For instance, soy protein nanoparticles contributed to increased release of resveratrol and soy β-conglycinin used as nanocarrier for curcumin, and both studies revealed an overall significant increase in stability and bioavailability (Liu et al., 2020, 2021).

## Challenges with nano-delivery methods

Despite the many advantages and opportunities NDS offer, several challenges still exist in their application in food product development. The safety of NDS is dependent on type of nanotechnology employed, application (*i.e*., the specific food, nutritional supplement or food contact materials), and other factors (*e.g*., temperature) (Buzby, 2010). While NDS improve the bioavailability, penetrability, and absorption potential, it also raises concern of toxicity (Livney, 2017). McClements & Xiao (2017) highlighted some vital mechanisms of nanotechnology toxicity in foods including interference with normal gastrointestinal function, accumulation of nanomaterials within specific tissues, alteration of release location, and interference with gut microbiota. Other authors (Jagtiani, 2022; Jain et al., 2018) have reported on the safety of nanomaterials, and subpar packaging performance highlighting the likelihood of nanoparticles moving from the from packaging material into food and their potential impact on consumer health. Jagtiani (2022) and Sahoo et al. (2021) expressed concern about heavy metals such as copper oxide, zinc oxide and silver released from nanomaterials, and their potential to increase intracellular ROS levels which damage DNA and enhance lipid peroxidation. Moreover, there are several questions related to the detrimental environmental impact. This include inability of certain nanomaterial to safely degrade in physiological and natural environments (Sampathkumar, Tan & Say, 2020). Earlier studies by Buzby (2010) discussed the potential negative impact of nano-silver used as a nanomaterial in food packaging on the environment. Sahoo et al. (2021) indicated that the use of titanium oxide as a nanomaterial in food packaging and its disposal can have an impact on people.

There are also concerns of health risks related to the delivery of bioactives and NDS (Reque & Brandelli, 2021), and little understanding of the toxicity of food-grade nanomaterials, and the need to determine their safety has been suggested (McClements & Xiao, 2017). Consumer perception, acceptance and knowledge of nanotechnology (Cummings et al., 2021) may also be an impediment to food application. The majority of respondents (67%) indicated that they had little knowledge and understanding of nanotechnology, nanomaterials, and nanoproducts, and about 42% responded they had little understanding of its effects on food (Arabeyyat, Jamaliah & Khalaf, 2022). Due to this low level of awareness, consumers are unable to make an informed evaluation of the potential risks to health and the environment (Ummi & Siddiquee, 2019).

The cost effectiveness of nanotechnology is another key challenge to their application in food (Yang & Duncan, 2021). Dadbin, Noferesti & Frounchi (2008) indicated that the cost per box of a product is likely to be higher if the food package is designed using nanomaterials (especially in the early stages of a product’s launch), estimated to be ≥10% of the product cost.

## Potential for commercialization and prospects

Toxins, viruses, and pesticides can detected using nano-based technologies, which also helps with tracking, tracing, and monitoring to ensure that food quality is maintained. However, most of these technologies are only limited to the laboratory and it is crucial that these technologies go beyond the confines of the laboratory and aid in resolving the problems facing society today.

Currently about two billion people globally are subjects of hidden hunger due to micronutrient deficiencies (Ofori et al., 2022). Siwela et al. (2020) reported that children under the age of five have a 43.6% and 11% incidence of iron and vitamin A deficiency, respectively, while children aged one to nine have a 45.3% risk of zinc deficiency. The micronutrients deficiencies have been attributed to loss of nutrients during processing and storage (Bajaj & Singhal, 2021; Sharma et al., 2020). Water-soluble vitamins and minerals are often lost during extensive food processing and storage (Martínez-Álvarez, Calvo & Gómez-Estaca, 2020). However, stability of these micronutrient in extensive processing and storage conditions have been achieved in laboratory settings through nano-based technologies (Gupta et al., 2019; McClements & Öztürk, 2021; Resende, Costa Lima & Reis, 2020; Sahafi et al., 2021). Building on the knowledge, commercializing this innovative approach could be instrumental in tackling hidden hunger. In Africa, South Africa, Egypt and Morocco have exhibited interest in commercializing nanotechnology in an effort to improve health and economic challenges (Mufamadi, 2019). In South Africa for instance, there is a national nanotechnology strategy that aims at supporting long-term nanoscience research and explorations of applications in sectors where nanotechnology could be beneficial. In a systematic review conducted by Harika et al. (2017) on four sub-Saharan African countries including South Africa, children aged 0 to 19 years were found to have anemia ranging from 25% to 53%, iron insufficiency (12–29%), vitamin A (14–42%), zinc (32–63%), and iodine (15–86%) deficiencies. In response to these issues, various policies such as diversifying one’s diet, taking supplements, and fortifying processed foods were implemented by the government but with limited success in alleviating micronutrient deficits (Siwela et al., 2020). However, with the current trends and policies in commercializing nanotechnology, it is expected that these health-related challenges could be alleviated or reduced. Future studies that examine the various approaches, implications, safety and commercializing of nano-based technologies including clinical trials, and increase awareness of the benefits and risks of nanomaterials will impact public acceptance (Sampathkumar, Tan & Say, 2020).

## Conclusion

Demand and market for functional foods are expected to grow substantially, and the potential for widespread application of nanotechnology such as nano-delivery systems. Nanomaterials facilitate the incorporation of bioactive ingredients into food products without altering their quality, texture, and other sensory attributes. This review discussed several nano-delivery systems, the benefits of protein-based NDS, and highlighted the limited research in their application. There is still the need to increase the limited public awareness of the benefits and risks of nanomaterials and further studies to support this growing segment of the food industry. Additionally, the need for as broader application and commercialization beyond the labs. Future studies should also expand beyond the use of whey protein, especially given the growing demand for vegan and plant-based food products. Moreover, increased efforts to identify approaches that can improve the safety of NDS will better facilitate the application of this technology in new functional foods.

## Supplemental Information

10.7717/peerj.14980/supp-1 Supplemental Information 1 PRISMA checklist.

## Additional Information and Declarations

### Data Availability

The following information was supplied regarding data availability: This is a literature review article.
